# A New Logit-Based Gini Coefficient

**DOI:** 10.3390/e21050488

**Published:** 2019-05-13

**Authors:** Hang K. Ryu, Daniel J. Slottje, Hyeok Y. Kwon

**Affiliations:** 1 Department of Economics, Chung Ang University, Seoul 156-756, Korea; 2 Department of Economics, Southern Methodist University, Dallas, TX 75275, USA; 3 Department of Political Science, Korea University, Seoul 136-701, Korea

**Keywords:** projection of share function, logit function, maximum entropy method, inequality measure, 62E17, 62P20, 91B15

## Abstract

The Gini coefficient is generally used to measure and summarize inequality over the entire income distribution function (IDF). Unfortunately, it is widely held that the Gini does not detect changes in the tails of the IDF particularly well. This paper introduces a new inequality measure that summarizes inequality well over the middle of the IDF and the tails simultaneously. We adopt an unconventional approach to measure inequality, as will be explained below, that better captures the level of inequality across the entire empirical distribution function, including in the extreme values at the tails.

## 1. Introduction

This paper introduces a new index of inequality that better captures information about the extreme values (the tail behavior) of observed distribution functions. The conventional approach to measuring inequality is to begin with an empirical distribution and to measure inequality in it by applying an index such as the Gini coefficient post facto (cf. Cowell [1] for an excellent discussion). We take the opposite approach; we begin with a given summary measure of inequality and then derive properties of the income distribution function (IDF) underlying it as expressed in the “shares” of the distribution. Ryu [2] was the first to use the “share function” as a vehicle to analyze inequality in empirical distribution functions. Following Ryu’s [2] work, Tanak et al. [3] and Rad et al. [4] cleverly apply the concept of the share function to an extended Gini measure (Tanak et al. [3]) and by introducing new specifications of the Lorenz curve (Rad et al. [4]), respectively. Here, we show that the income share ratio of the richest group to the poorest group of a given observed empirical income distribution can be derived accurately using the income distribution function derived from our new measure. We call our new measure the logit-based Gini coefficient (LBGC). 

Several statisticians have examined the issue of measuring and describing inequality in statistical distribution functions (cf. Arnold and Sarabia [5], Arnold, Castillo, and Sarabia [6], Giorgi [7], Giorgi and Gubbiotti [8], and Nygard and Sandstrom [9,10] among others, for a broad discussion of many of these issues). For traditional income inequality measurement, the Gini coefficient is by far the most widely used index even though other measures exist. The seminal work of Lerman and Yitzhaki [11] and Yitzhaki [12] demonstrated that the Gini coefficient can be derived from the first moment of the observed income shares for a given batch of income data. During this filtering process, the Gini in effect only projects a linear trend and throws out the remaining information contained in the observed share values. This information loss problem can be remedied, and the major contribution of this paper is that it introduces one way to do so by projecting the income shares with the logit function. 

The choice of a weighing function in the projection uniquely determines the inequality measure. The range of the logit function varies from minus infinity to plus infinity and it can detect and project changes in the very small shares of the IDF’s poorest group’s income shares and the IDF’s richest group’s income shares.

This approach can be applied to higher moments in an IDF as well. In Appendix B, projections with other moments such as the second moment, third moment, square root moment, and exponential moment are explained. Each of these other forms is a legitimate inequality index. However, the performance of these derived functions from their moments was not quite satisfactory and each alternative measure more or less provides the same level of information as contained in the original Gini coefficient. The reason for these relatively poorer performances comes from the finite range of the weighting function used in these other projections. The ranges of the first moment, second moment, and square root moment are restricted between zero and one. As noted above, Appendix B of this paper discusses the details of these other measures, but this paper focuses on the LBGC measure.

Because this paper introduces a new LBGC summary inequality measure, it is necessary to check to confirm the new inequality measure possesses desirable properties of an income inequality measure. These well-known properties are listed in Appendix A where it is also shown that the properties are satisfied by the new measure. For practical purposes, we also proffer the following properties, which are shown to be satisfied. (1) The income inequality measure should be sensitive to the movements of income shares of the poorest and richest groups. (2) The underlying IDF can be derived directly from the given summary measure and it is hoped the estimates will be accurate at all ranges of income groups. (3) If an economic interpretation can be given to the definition of the summary measure, it provides guidelines indicating how best to use the measure.

In the next section, we discuss the linear projection method utilized in applying the well-known Gini coefficient. The underlying income distribution function is derived by the maximum entropy method subject to the given Gini coefficient. In Section 3, we introduce the new summary inequality measure and the LBGC measure, and the corresponding underlying distribution function is derived. In Section 4, Current Population Survey data are utilized to show the usefulness and limitations of analysis made with the Gini coefficient and with the new LBGC measure. Section 5 concludes the paper.

## 2. Motivation for Our Analysis

To begin, we define an income inequality measure as a projection of the income share function with some weighting function. The Gini coefficient is derived by utilizing a linear weighting function, Theil’s entropy measure utilizes the logarithm of the share as a weighting function, and the new LBGC measure is derived by imposing a logit function as the weighting function. To understand our introduction of the logit function, consider the following: suppose the income observations have a lognormal distribution. This means the logarithm of the income observations is normally distributed. It is well known that the poorest segments of the population across virtually all countries have (as groups) income shares very near to zero. Of course, the logarithm of a value approaching zero is a large negative number. Concomitantly, the normal distribution possesses a point of “high contact” in classical statistics, meaning the poorest segment in the left tail has a very small accumulated probability. With the inversion of the accumulated normal distribution, a small far left tail above corresponds to a small *z* value (*z* is near zero in our paper and is explicated below). However, the accumulated normal distribution does not have an explicit inverse functional form; if it did, it could have been used as a projection function. Therefore, we use the logit function because it does have an explicit functional form and the shapes of the probit and logit functions are similar.

Therefore, we use the logit function as a projection function in (13) below, because both the logit function and share function become very large for *z* near one. For the left tail of the distribution, when *z* approaches zero, the logit approaches negative infinity but the share function approaches zero. Therefore, based on the logit projection upon the share function in (13), entropy maximization produces a share function (17). The logarithm of this share function includes a logit form and converges to a lognormal distribution. The difference, again, is that we now have an explicit functional form of share function based on the logit projection because we cannot derive an explicit functional form for the probit case. The imposition of other weighting functions will also yield other legitimate inequality measures. In Appendix B, a second moment, a third moment, a square root moment, and exponent moment are used as weighting functions. We present them for the reader’s consideration only. The LBGC measure describes inequality in the overall IDF better, but these other measures are interesting. 

### 2.1. Derivation of the Gini Coefficient with a Linear Projection Function

In order to show that the usual derivation of a Gini approximation neglects all the remaining information contained in observed share values, one can first introduce and then expand the share function with a Legendre polynomial series. Suppose we have an IDF (income distribution function) and we have income observations for 100 individuals $q_{1},q_{2},\ldots ,q_{100}$. We can plot a histogram with these 100 income observations. Alternatively, if these observations are normalized, the income share of the *i*-th person is $s_{i}=q_{i}/(q_{1}+q_{2}+\cdots +q_{100})$. The share variable is a random variable. As an example, when a dollar bill falls from the sky, the probability it will land on the *i*-th person is $s_{i}$. Different people have different capabilities of picking up the dollar bill as it falls from the sky. For a continuous share function s(z)for0≤z≤1, the income shares of the poorest and richest are

$$s_{1}=\int_{0}^{0.01}s(z)dz\;\mathrm{and}\;s_{100}=\int_{0.99}^{1}s(z)dz.$$

We can show that the Gini coefficient can be derived from the first moment of the share function. Suppose n is the number of persons and assume *n*-1 is approximately equal to *n*. Using the Lorenz curve definition,

$$Gini=1-\frac{2}{n-1}\left(n-\frac{\sum_{i=1}^{n}iq_{i}}{\sum_{i=1}^{n}q_{i}}\right)=1-\frac{2}{n-1}\left(n-\frac{\sum_{i=1}^{n}is_{i}}{\sum_{i=1}^{n}s_{i}}\right)\approx 1-2(1-\mu )$$

The first moment becomes $\mu =\sum_{i=1}^{n}is_{i}/n\approx \int_{0}^{1}zs(z)\mathrm{dz}$.

**Figure 1:**
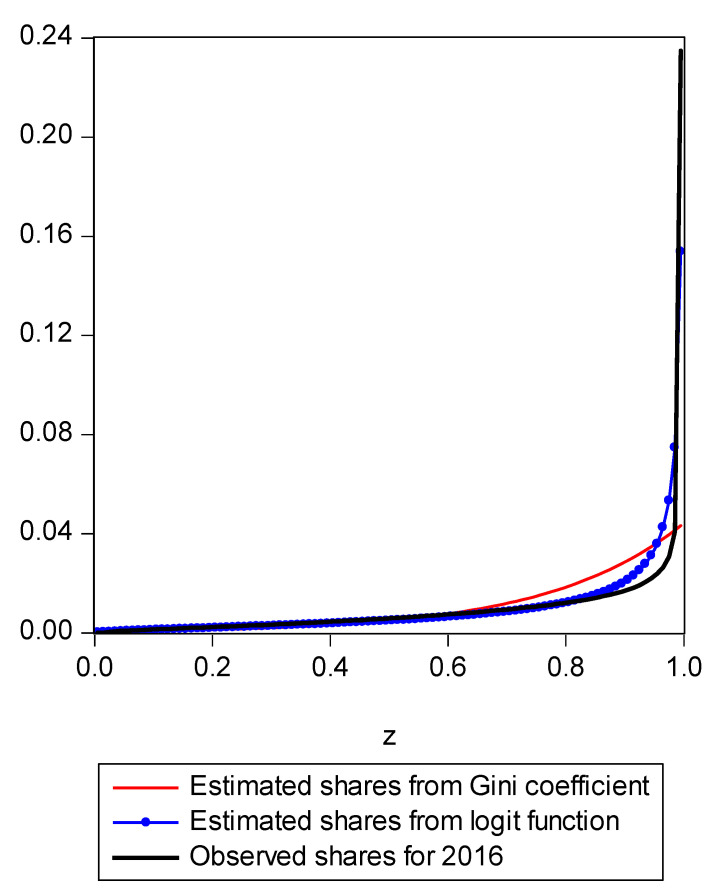
LBGC estimated shares and Gini estimated shares.

The share function is related to the underlying income distribution by virtue of the fact that a Lorenz curve underlies (can be constructed from) any income distribution function and the first derivative of the Lorenz curve is the share function. Conversely, if one were to accumulate the share function piecewise, this accumulation would become a Lorenz curve. Tanak et al. [3] and Rad et al. [4] also recognized the issue of the Gini index being a poor indicator of movements in the tails of the empirical distribution and how using entropy and the attendant share function can be specified in a way that can more accurately capture the tail behavior of the observed distribution. It should be noted that Gastwirth [13] dissented from this view and showed a situation where the Gini coefficient is more sensitive to changes in the lower and upper parts of the distribution than in the middle. However, our point is not about the sensitivity of the Gini coefficient per se to changes in an income distribution. Rather, the income shares of the top 5% of incomes can be reproduced very accurately by our new LBGC measure whereas those of the Gini were not very good. We are concerned less with the direction and sensitivity of the reproduced results by the Gini coefficient but rather about the magnitude and direction of reproduced results relative to those reproduced by the LBGC measure. Figure 1 also illustrates the relatively poorer performance of the Gini for the wealthiest segment of the distribution. The generalized Gini index is defined as (see papers of Rad et al. [4] at p. 2911 and Tanak et al. [3] at p. 281)

$$G_{\upsilon }=1-\upsilon (\upsilon -1)\int_{0}^{1}{\left(1-p\right)}^{\upsilon -2}L_{x}(p)dp,\;\upsilon >1$$

where $L_{x}(p)$ is the Lorenz curve of population proportion p. If υ=2, the generalized Gini index is equivalent to the classical Gini index. The primary difference between the generalized Gini index from the classical Gini index is the following. The former uses an additional curvature parameter υ but the “cost” is the loss of simplicity in describing income inequality with a single measure (either the Gini, or Theil, or others). For different data sets, a researcher would need to select different values of υ. In our work, the income share functions can be derived based on a given set of conditions using the maximum entropy method. As will be shown below, our paper is based on a procedure that only requires knowledge of a single parameter Equation (13) or equivalently Equation (16), whereas the procedures of Rad et al. [4] and Tanak et al. [3] require knowledge of both υ and $G_{\upsilon }$. Therefore, the derived share functions in their work become functions of z and υ, instead of just on *z* as in our work. Arfken [14] and Milne [15] present a good explanation of the orthonormal basis method and the use of Legendre polynomials.

(1) $$s(z)\approx s_{N}(z)=\sum_{n=1}^{N}a_{n}P_{n}(z)$$

The Legendre polynomials are the following for 0≤z≤1:

(2) $$\begin{array}{l} P_{0}(z)=1\\ P_{1}(z)=\sqrt{3}\left(2z-1\right)\\ P_{2}(z)=\sqrt{5}\left(6z^{2}-6z+1\right)\\ P_{3}(z)=\sqrt{7}\left(20z^{3}-30z^{2}+12z-1\right)\\ P_{4}(z)=\sqrt{9}\left(70z^{4}-140z^{3}+90z^{2}-20z+1\right)\\ P_{5}(z)=\sqrt{11}\left(252z^{5}-630z^{4}+560z^{3}-210z^{2}+30z-1\right) \end{array}$$

An orthonormal sequence satisfies

(3) $$\int_{Z}P_{n}(z)P_{m}(z)dz=\delta_{nm},\;n,m,=0,1,2,\cdots$$

where $\delta_{nm}=1$ if n=m and zero otherwise. The parameters of (1) can be found with

(4) $$a_{m}=\int P_{m}(z)s_{N}(z)dz=\int P_{m}(z)\left[\sum_{n=1}^{N}a_{n}P_{n}(z)\right]dz$$

Lerman and Yitzhaki [11] argued that knowledge of the Gini coefficient is equivalent to the knowledge of the first moment. This assertion is proved above. They stated in their paper, “Using the regression coefficient yields a general graphical interpretation of the Gini. Consider a case of an honor guard of soldiers in which the soldiers are ordered by height from the shortest to the tallest and are equidistant from adjacent soldiers. Assume that the entire length of the honor guard is a distance of 1. Using this ordering and running a regression of height against the soldier’s position will yield a regression line that passes through the mean value (height) at the mean rank (or the 50th percentile). The slope of this regression line is the value of the absolute Gini times a constant.” (Lerman and Yitzhaki [11], p. 365)

If the Gini coefficient utilizes only the first moment,

(5) $$\mu_{1}=\int zs(z)\mathrm{dz}=\frac{1+\mathrm{Gini}}{2}\approx \int zs_{N}(z)\mathrm{dz}=\int z\sum_{n=1}^{N}a_{n}P_{n}(z)dz$$

Use $z=\frac{P_{1}(z)}{2\sqrt{3}}+\frac{P_{0}(z)}{2}$.

(6) $$\mu_{1}\approx \int \left[\frac{P_{1}(z)}{2\sqrt{3}}+\frac{P_{0}(z)}{2}\right]\sum_{n=1}^{N}a_{n}P_{n}(z)dz=\left[\frac{a_{1}}{2\sqrt{3}}+\frac{a_{0}}{2}\right]$$

Therefore, the Gini coefficient uses only a linear approximation of the share function (1) and it neglects all the remaining information contained in the raw data. To see why this is so, consider that we can derive the same Gini coefficient value even if we assume the raw data generating function is

(7) $$s^{*}(z)=a_{0}P_{0}(z)+a_{1}P_{1}(z)$$

where the parameters $a_{0}$ and $a_{1}$ are the same as those of (1).

### 2.2. Derivation of a Share Function from the Given Gini Coefficient

If raw income data are unavailable, information about the underlying income distribution function can still be uncovered by extracting information from the summary index that is available, such as that contained in the given Gini coefficient. This paper now explains how that is done. This section begins with a review of Ryu and Slottje [16,17], which is the first step towards our final objective of presenting more information-rich inequality measures. Solving an entropy maximization problem of Shannon [18] and Ryu [2] subject to the given first moment:

(8) $$Max_{s}W\equiv -\int s(z)\log s(z)dz$$

satisfying

(9) $$\int zs(z)dz=\mu_{1}$$

where s(z) is the share function of a person located at z. The Lagrangian method produces

(10) $$s_{Gini}(z)=\exp\left[a+bz\right]=\left[\frac{b}{e^{b}-1}\right]\exp[bz]$$

where the normalization condition of the share function is used to remove a. Now the first moment condition (5) produces

(11) $$\mu_{1}=\left[\frac{b}{e^{b}-1}\right]\int_{0}^{1}z\exp[bz]dz=\frac{1+\mathrm{Gini}}{2}$$

Since the integration is a function of *b*,

(12) $$\mu_{1}\equiv -\frac{1}{b}+\frac{e^{b}}{e^{b}-1}=\frac{1+\mathrm{Gini}}{2}$$

Then b approaches zero if the Gini = 0 and b approaches infinity if the Gini = 1. Since the center part of (12) is a monotonic increasing function of b, a given Gini coefficient will uniquely determine b and the income share function s(z). 

## 3. Derivation of the LBGC and the Corresponding IDF

Suppose we define a summary measure with the projection of a share function s(z) with a weighting function ψ(z), i.e.,

(13) $$\phi =\int_{0}^{1}\psi (z)s(z)dz$$

Suppose the logit function is defined as

(14) $$\psi (z)=\mathrm{logit}(z)=\log\left(\frac{z}{1-z}\right)$$

The projection of a share function with the above logit function is

(15) $$\phi =\int \log\left(\frac{z}{1-z}\right)s(z)\mathrm{dz}$$

For the uniform distribution with equal shares or for the extremely unequal shares where only one person has all the income, ϕ will have the values

$$B=\int_{0}^{1}\log\left(\frac{z}{1-z}\right)dz\;\mathrm{or}\;\log(2n)$$

where n is the sample size. Now, define the inequality measure LBGC as

(16) $$LBGC=\frac{\phi -B}{\log(2n)-B}$$

Then LBGC will be zero for the extremely equal or uniform distribution, and LBGC will be one for the extremely unequal distribution, where (say) all the mass is at one point in the tail.

The Lagrangian method of (8) subject to ϕ and normalization produces

$$L=\int s(z)\log s(z)dz+\lambda \left[\int s(z)dz-1\right]+\gamma \left[\int \log\left(\frac{z}{1-z}\right)s(z)dz-\phi \right]$$

Maximization with respect to s(z) produces

$$\log s(z)+1+\lambda +\gamma \log\left(\frac{z}{1-z}\right)=0$$

$$s(z)=\exp\left[-\left(1+\lambda +\gamma \log\left(\frac{z}{1-z}\right)\right)\right]$$

(17) $$s_{L}(z)=C(\gamma ){\left(\frac{z}{1-z}\right)}^{\gamma }$$

For the chosen γ, C(γ) is found with normalization. Choose γ such that the estimated logit moment of (15) will be the same as the observed logit moment.

$$\phi_{\gamma }=\int \log\left(\frac{z}{1-z}\right)C(\gamma ){\left(\frac{z}{1-z}\right)}^{\gamma }\mathrm{dz}$$

Suppose percentile share data are given for an economy. Then one can summarize inequality with (15) and get $\phi^{*}$. For various values of γs, we can find $\phi_{\gamma }$ by numerically adding values at the midpoint of z = [0,0.01], …, [0.99,1.0]. Since $\phi_{\gamma }$ is an increasing function of γ, a unique $\gamma^{*}$ is found corresponding to $\phi^{*}$. 

## 4. Applications

To demonstrate the usefulness of this new approach, we utilize Current Population Survey (CPS) data for the years 2000 through 2016. Because the Gini coefficient and the LBGC are both inequality measures that are in fact functions of underlying income shares, an underlying share function can be uniquely determined for each (as was shown in Equation (10) for the Gini and Equation (17) for the LBGC). These underlying share functions afford us the opportunity to directly compare how the Gini and LBGC measures perform in fitting actual income distribution share data. That is, we can compare apples to apples by seeing how various expected shares from the Gini and LBGC compare to actual shares of an actual empirical distribution, across various parts of the empirical distribution. Using CPS data divided into income centiles, we can examine directly how the two measures perform in fitting actual observed shares over the entire actual empirical distribution, divided into centiles (100th percentiles). Concomitantly, we can also perform the exercise by isolating various regions of the actual observed centile shares to focus on the poorest groups and richest groups. Recall that our hypothesis is that while the Gini is an excellent intuitive measure of inequality, it doesn’t perform as well in capturing the level of inequality inherent in the tails because it loses information as it is “first moment focused”; since the empirical distribution tails may contain extreme values and the share function derived from the Gini coefficient may not detect that the tail has extremely high or low values, the Gini index will not accurately capture that fact. However, because the LBGC measure captures information over the entire distribution and the share functions underlying it take into account all values in the distribution including in the extreme tails, the share function derived from the LBGC is quite accurate. To see this, consider Figure 1.

We used Shazam version 11 for computer calculations and Eviews 10 for plotting the graphs. 

**Figure 2:**
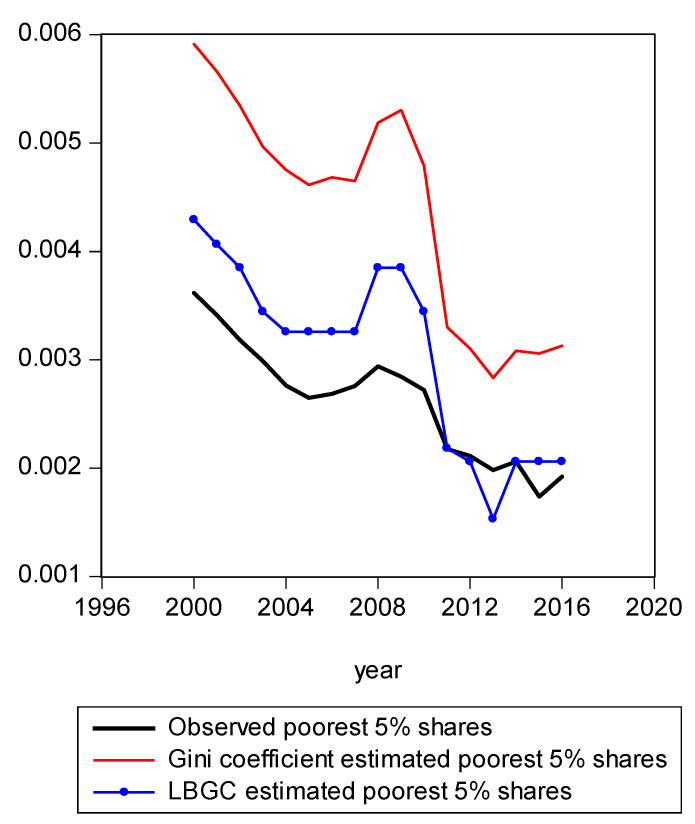
Poorest 5% shares estimated from Gini and LBGC.

**Figure 3:**
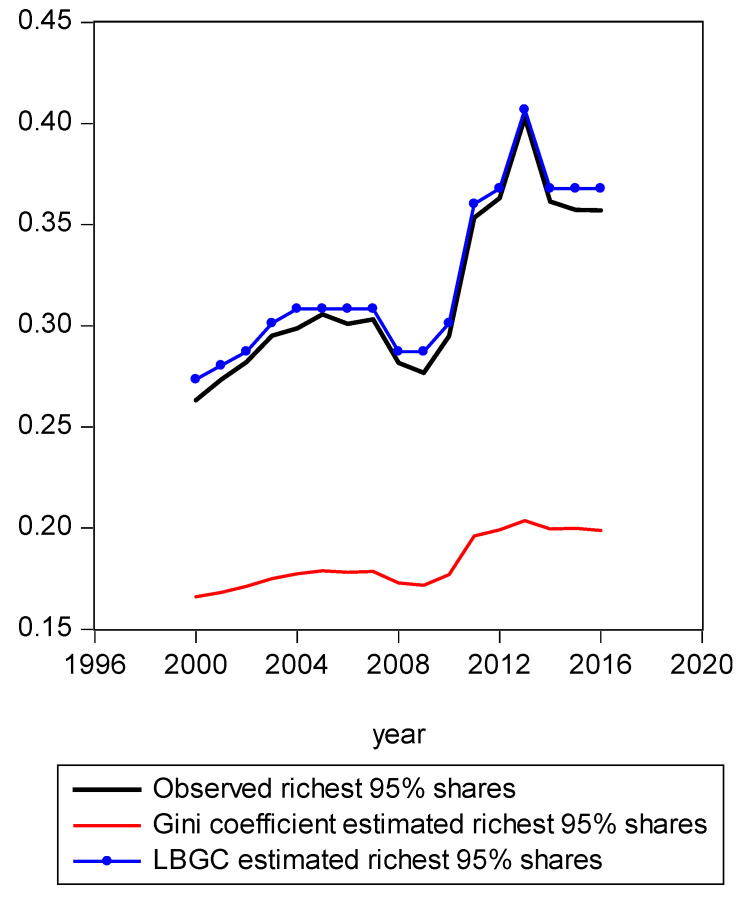
Richest 95% shares estimated with Gini and LBGC.

In Figure 1, we present a mapping of the estimated Gini-coefficient-derived shares and the estimated logit-function-derived shares against the actually observed centile income shares for 2016, for the entire observed empirical distribution function. The black line represents the percent of actual observed income held by each centile share against the shares predicted by the Gini (red line) and logit (LBGC, the blue line). A perfect fit would mean the lines lie on top of each other as one moves from lower income centiles to successively higher ones. As can be seen from Figure 1, it is evident that the Gini estimates do not perform well at the very highest income centile shares while the LGBC function does a very good job of tracking with the highest centiles of actual income shares in 2016. While not as evident from the graph in Figure 1, the lower tails of the actual empirical distribution are also approximated better by the LBGC functional than by the Gini coefficient. Figure 2 illustrates that when the poorest 5% income group shares are examined, having been derived from both the Gini coefficient and the LBGC measure, and are then compared with the observed shares for the CPS data for the years 2000–2016, the LBGC-measure-derived shares more accurately approximate the actual centiles and also capture movement across the lowest centiles of income shares more accurately. As can be seen by viewing the blue line (LBGC) and the red line (Gini), it is evident that estimation with the LBGC-based shares is more precise in comparison to the Gini coefficient as the blue line is closer to the actual observed shares represented in the black line. In Figure 3, the richest 5% of income group shares are compared in the same way as in Figure 2. The richest 5% of income shares are estimated well using the LBGC-derived shares as the blue line lies almost on top of the black line. The Gini-derived shares fit relatively poorly. The purpose of these graphs is to show precisely these facts. In fact, the same holds true across all of the empirical distributions for all of the years for which we have data. 

To summarize in an intuitive manner how the shares derived from the two inequality models performed against each other for the period from 2000 to 2016, we examined graphically how observed income shares have changed from the CPS data over time and compared graphically the fit of those to the estimated shares from our underlying projections. Of course, it is possible to assess the relative fit of the shares derived from our inequality models more explicitly. To do so, we calculate the sum of squared residuals. We found that the sum of squared residuals (RSS) produced by various models produced very clear results. For example, recalling that the estimated share based on the observed Gini model is presented in Equation (10) and the LBGC-measure-derived share is presented in (17), we can dispense with the heuristic graphical presentations to show the actual fits for each model explicitly. The RSS is a standard fit criterion,

(18) $$RSS_{Gini}(t)={\left[{\mathrm{Observed}\;\mathrm{share}(t)}_{i}-{\mathrm{Gini}\;\mathrm{Estimated}\;\mathrm{share}(t)}_{i}\right]}^{2}$$

**Figure 4:**
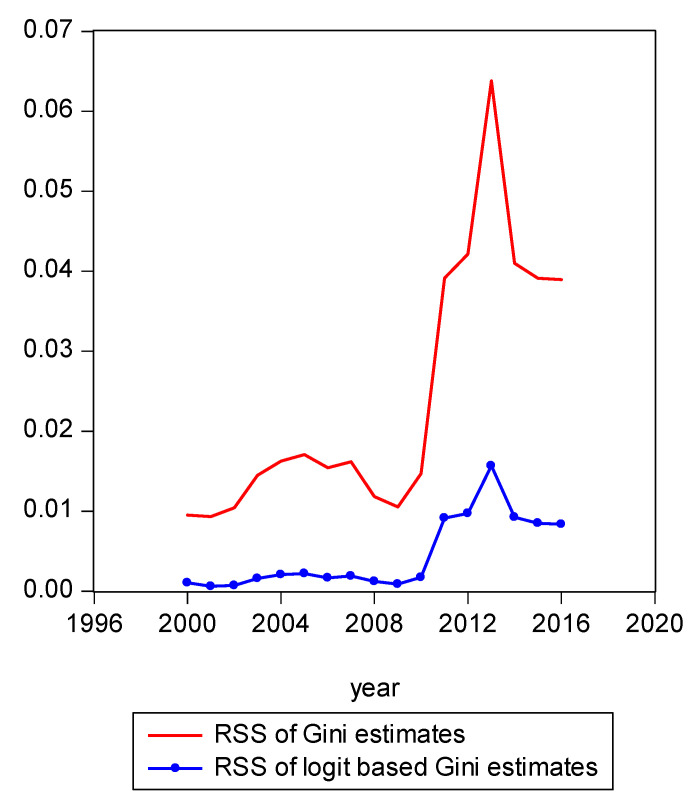
Comparison of residual sum of squares.

As Figure 4 illustrates, one can see that year 2013 was somewhat of an anomalous year with more income inequality than prior years or years that followed. The share of income of the richest 1% of earners was 29% in 2013. In comparison, the richest 1% share of income in 2015 was 23%. Figure 4 shows the LBGC model has an absolute advantage relative to the Gini model in that it yields a much lower RSS. It is frequently the case that a policymaker does not have access to the underlying data and only has access to or wants to focus on a single measure or indicator of inequality. In those instances, the logit variant of the Gini might be seen as most useful in gauging how overall inequality has changed over time.

## 5. Conclusions

This paper introduced a new income inequality measure as a projection of the share function with a particular weighting function. The choice of weighting function uniquely determines the summary measure. By choosing a logit function as a weighting function, a summary measure is determined which we labeled the LBGC measure. The underlying income distribution function (IDF) is estimated using the maximum entropy method subject to the given LBGC. The movements of extreme income share changes are described well with the estimated IDF. Movements of the vanishing middleclass income share are also detected well with the LBGC measure. The Gini coefficient moves in a parallel path with the LBGC and it gives good directional information on worsening inequality but it could not provide quantitative information on the rapidly increasing income share of the richest group. The particular shape of the logit function is sensitive to movements in income shares of the poorest and richest groups. This logit measure and the derived share function provide a more useful tool for policy makers who actually have to be concerned with how (say) tax policy will affect the poorest and richest segments of a society.

## Figures and Tables

**Figure A1:**
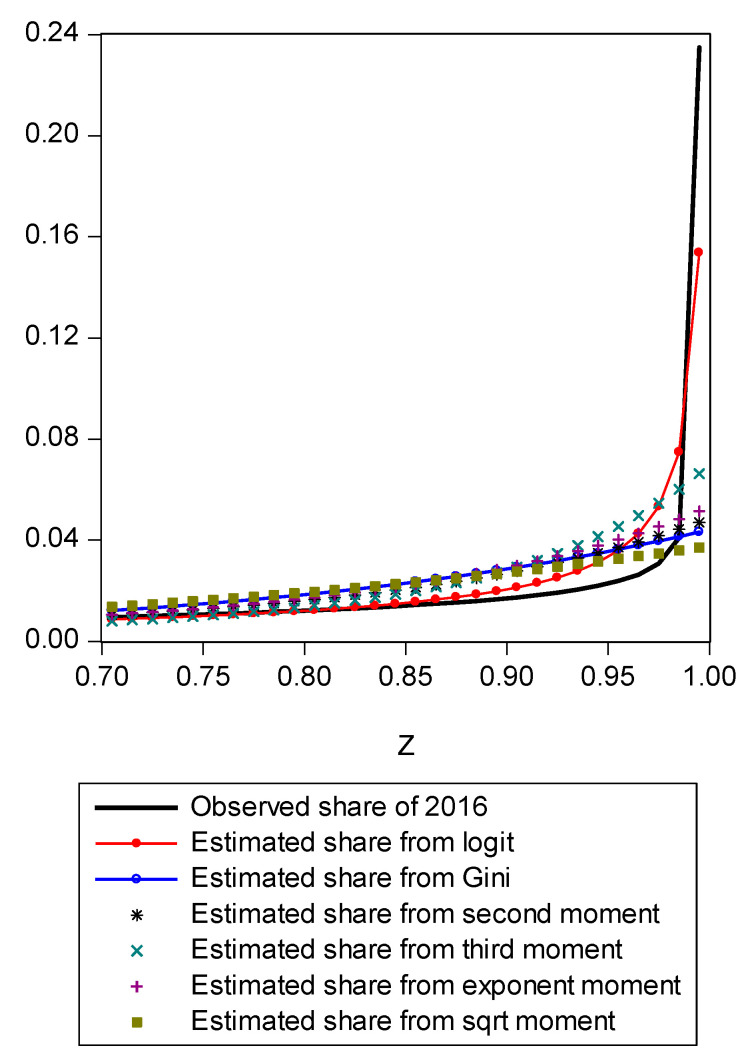
Estimated shares from various moments.

**Figure A2:**
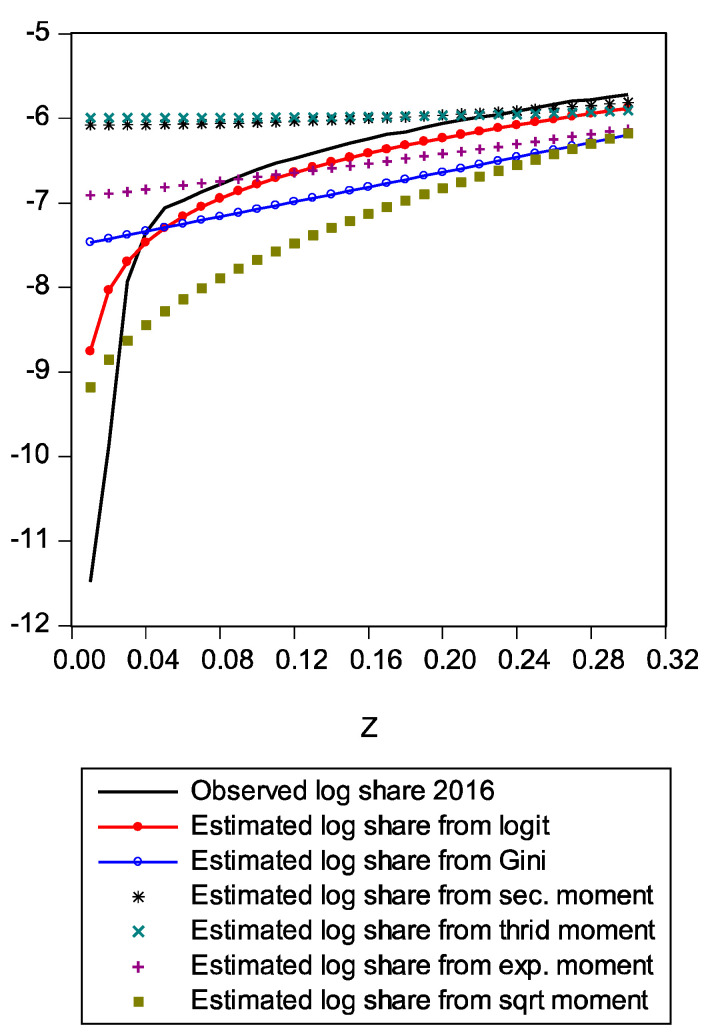
Comparison of log shares from various moments.

**Figure A3:**
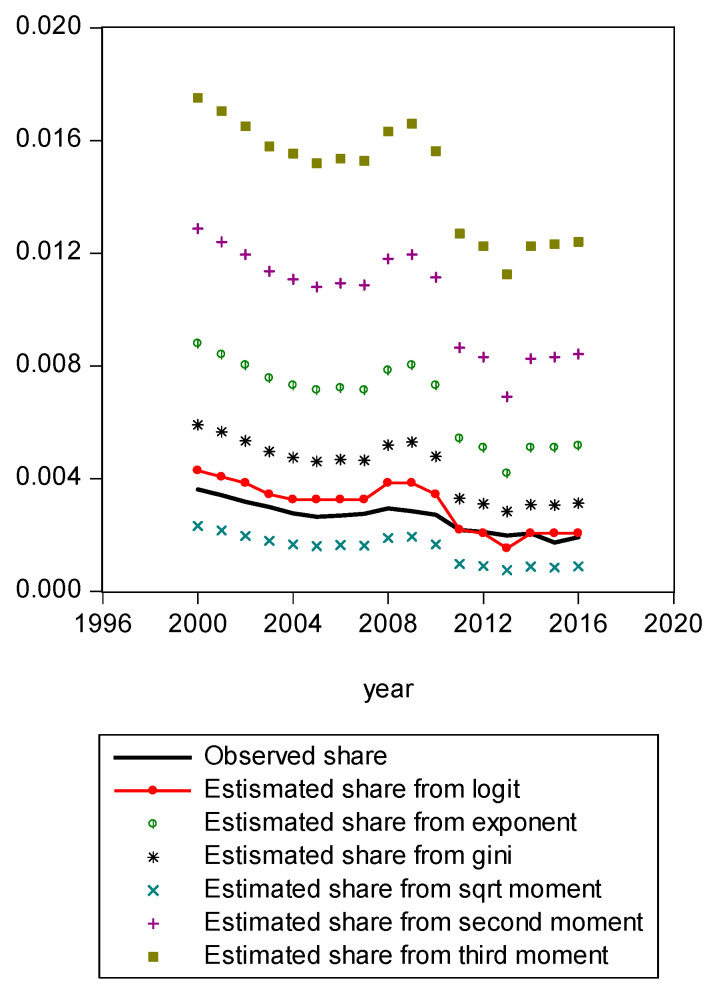
Comparison of various methods for bottom 5% share.

**Figure A4:**
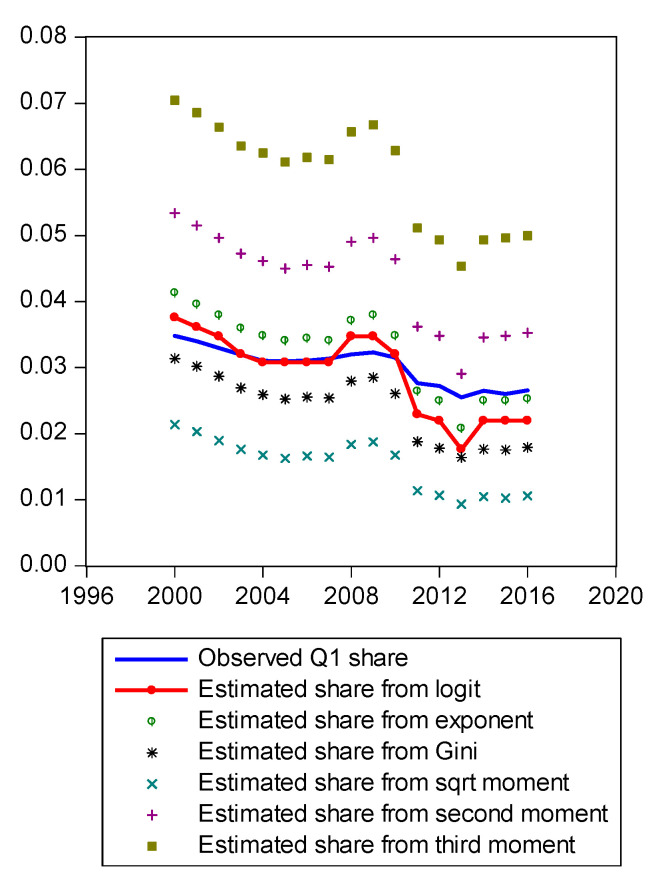
Comparison of various methods for Q1 shares.

**Figure A5:**
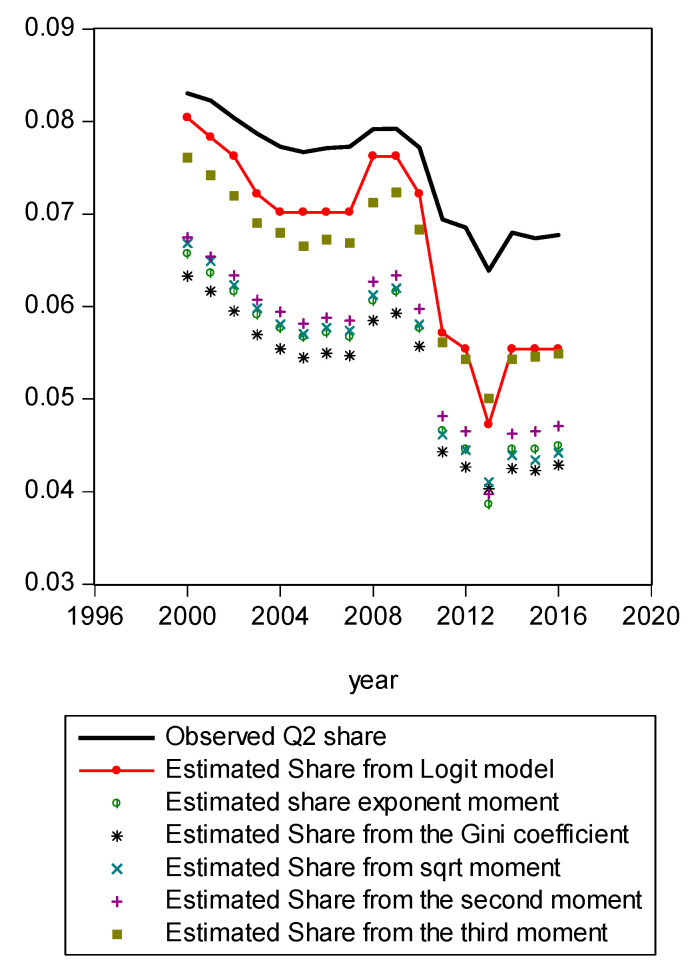
Comparison of various methods for Q2.

**Figure A6:**
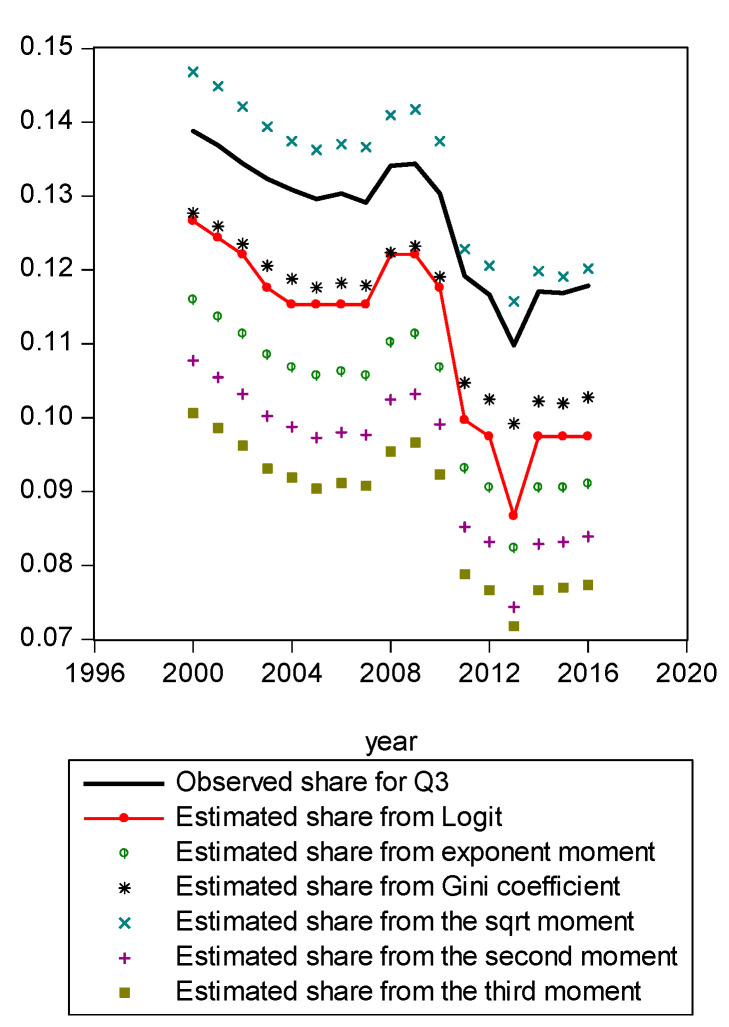
Comparison of various methods for Q3.

**Figure A7:**
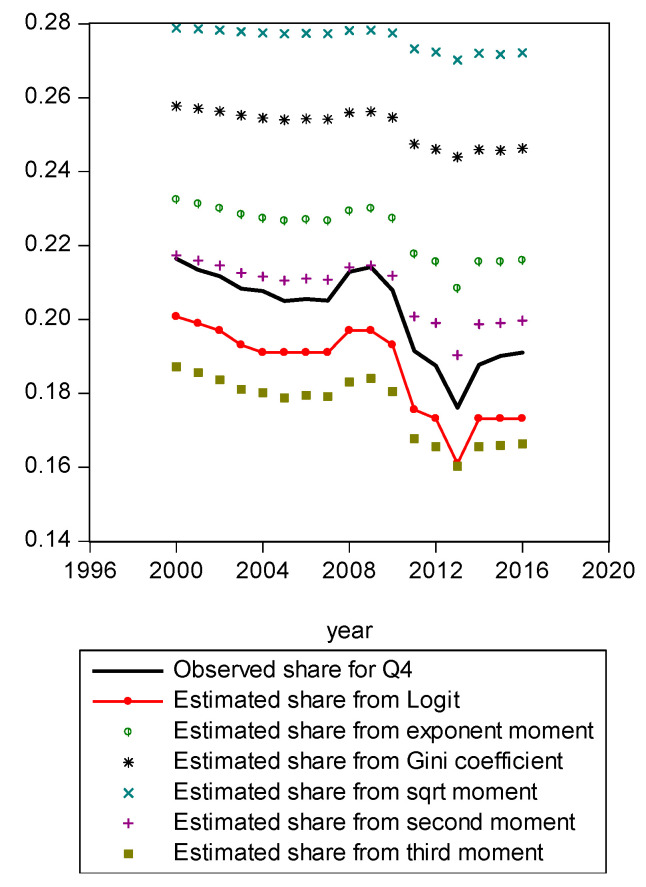
Comparison of various methods for Q4.

**Figure A8:**
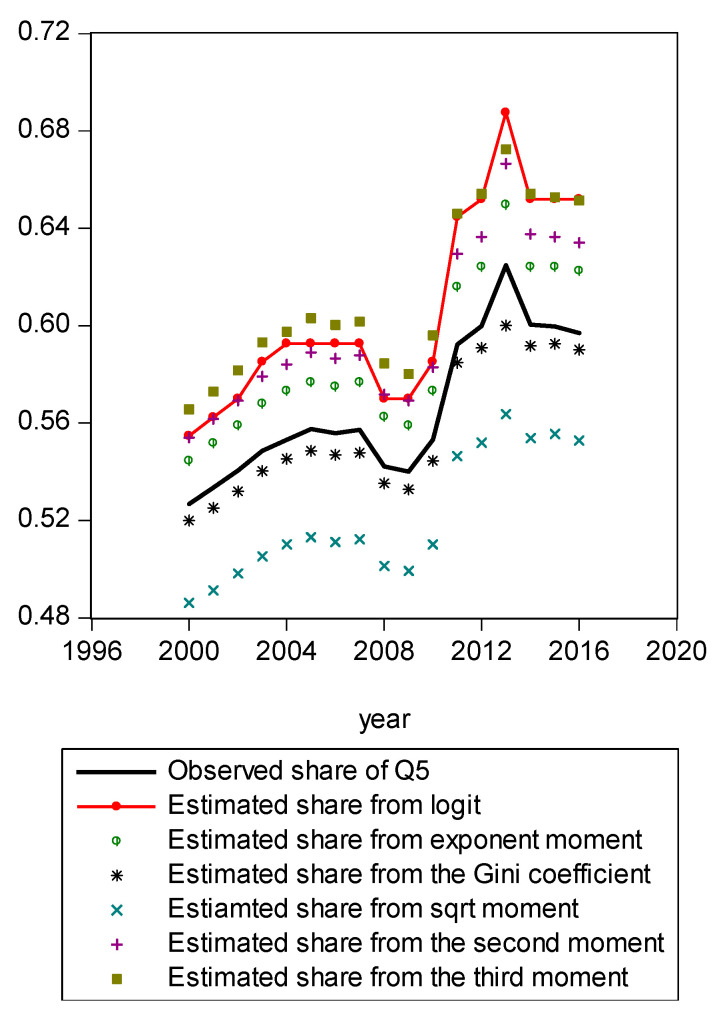
Comparison of various methods for Q5.

**Figure A9:**
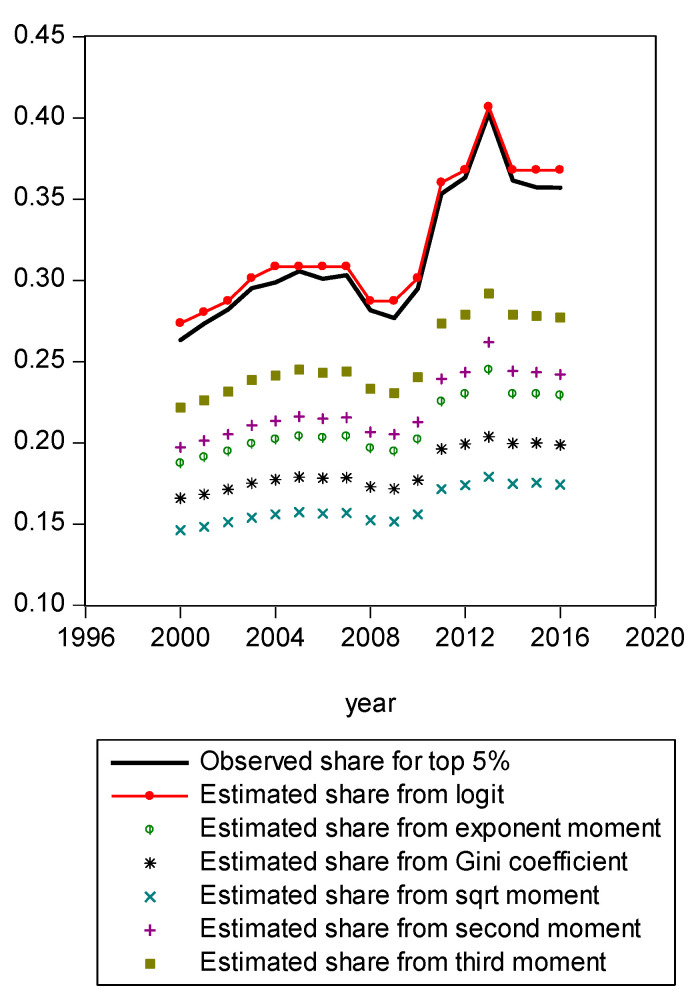
Comparison of various methods for top 5% share.

**Figure A10:**
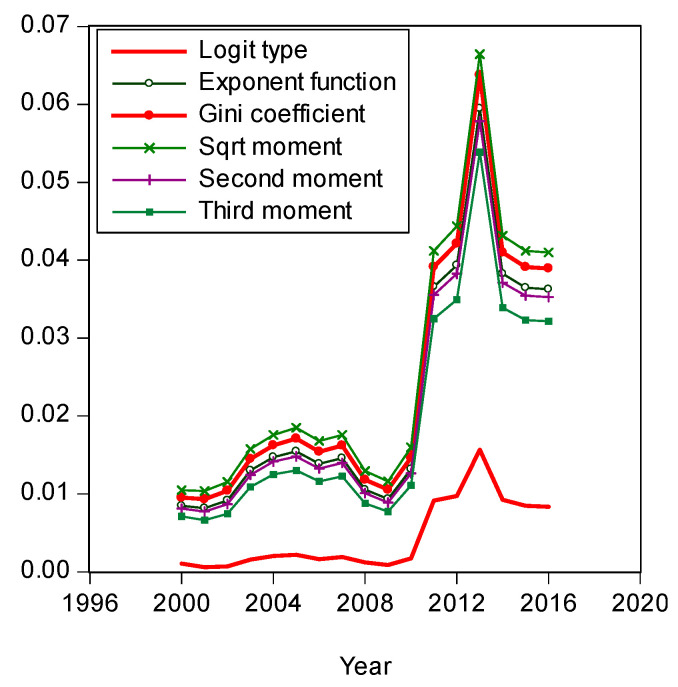
Comparison of Residual Sum of Squares.

## Appendix A. Desirable Properties of an Income Inequality Index

This list is a collection whose individual properties are discussed in many places, including Cowell [1], Sen [19], and Ryu and Slottje [17], among others. They are enumerated in an excellent appendix by Sen and Foster, contained in Sen [19].

Given the inherent difficulty in describing the characteristics of an entire IDF with one number, the following properties for an income inequality index are ***desirable.*** Professor Sen’s seminal book in its third edition in 2003 added an appendix with Professor James Foster that rigorously laid out these axioms:

**Anonymity or symmetry**

The inequality measure should not depend on how individuals in an observed distribution are labeled (cf. Sen and Foster [19], Appendix A3.2).

**Scale independence or homogeneity**

As Cowell ([1], p. 63) notes, the measured inequality of the slices of the cake should not depend on the size of the cake. This property says that if (say) every person’s income in an economy is increased by some constant, then the overall metric of inequality should not change.

**Population independence**

Similarly, the inequality measure should be independent of the level of population. Cowell ([1], p. 63) notes the inequality of the cake distribution should not depend on the number of cake receivers.

**Transfer principle**

The Pigou–Dalton, or transfer principle, states, in its weak form, that if income is transferred from a rich person to a poor person while still preserving the order of income ranks, then the inequality measurement should not increase (cf. Cowell [1], p. 62).

**Non-negativity**

The inequality index *I(y)* must be greater than or equal to zero (cf. Ryu and Slottje [17] and Sen and Foster [19], Appendix A3.3).

**Egalitarian zero**

The index *I(y)* is zero when everyone has the same income, meaning when all values y*_i_* are equal (cf. Ryu and Slottje [17] and Sen and Foster [19], Appendix A3.2).

**Bounded above by maximum inequality**

The index *I(y)* attains its maximum value of one, reflecting the maximum level of inequality (all $y_{i}$ are zero except one) (cf. Ryu and Slottje [17] and Sen and Foster [19], Appendix A3.1).

In this paper, we utilize the Gini coefficient and introduce a new inequality measure based on the logit type function. Since we use only 100 percentile income shares, anonymity, scale independence, and population independence are satisfied for the summary measure used in this paper. Non-negativity, egalitarian zero, and “bounded by maximum inequality” are also satisfied for the summary measure (LBGC) used in this paper. The remaining property is the transfer principle. The projection function, the logit function, is a monotonic increasing function. Therefore, if a small portion of income is transferred from a rich person to a poorer person, then the projection with a monotonic increasing function will decrease and inequality will decrease. Thus, the transfer principle is satisfied.

## Appendix B. Various Weighting Functions for Inequality Measures

In this paper, we introduced a projection of a share function with a weighting function of a logit function. This functional form was one of several moment-based weighting function candidates considered. Suppose we label the inequality measure derived from the second moment as *G_2_*, the inequality measure derived from the third moment as *G_3_*, the inequality measure derived from the square root moment as *G_sqrt_*, and the inequality measure derived from the exponential moment ($\mu_{\exp}$) as $G_{\exp}$. The exact meaning of these derived Ginis will be explicated below. However, at this juncture, we simply want to note that each of these “derived Ginis” are derived from the underlying share function and subsequently, we can derive a share function (s_2_) using the maximum entropy method from the second moment ($\mu_{2}$), derive a share function (s_3_) from the third moment ($\mu_{3}$), derive a share function (s_sqrt_) from the square root moment ($\mu_{sqrt}$), and derive a share function (s_exp_) from the given exponent moment $\mu_{\exp}$.

Once these derived share functions are determined, these approximated shares will be compared to the observed shares used to calculate the original Gini. Finally, using US CPS data for the period from 2000 through 2016, we can compare the performance of the Gini, *G_2_*, *G_3_*, $G_{sqrt}$, $G_{\exp}$, and LBGC of (16) with respect to movements of the income quantiles over time by examining changes in the observed IDF by honing in on the poorest 5% shares, Q1, Q2, Q3, Q4, Q5, and the richest 5% income shares of the observed shares. This exercise will allow us to glean more information about how movement within the observed IDF (and its attendant summary Gini coefficient) has fluctuated over time. We will also examine our proffered measures with respect to desirable properties that an income inequality index should possess, such as symmetry, scale independence, population independence, transfer principle, non-negativity, egalitarian zero, and bounded above by maximum inequality. The examination will be performed for *G_2_, G_3_*, $G_{sqrt}$, $G_{\exp}$, and LBGC and the proofs that these measures satisfy desirable properties that any inequality measure should possess can be explained with the same logic listed in Appendix A above.

### Appendix B.1. Derivation of an Inequality Measure and Corresponding Share Function from the Given Second Moment

If only the second moment is utilized,

(A1) $$\mu_{2}=\int z^{2}s(z)\mathrm{dz}\approx \int z^{2}s_{N}(z)\mathrm{dz}=\int z^{2}\sum_{n=1}^{N}a_{n}P_{n}(z)dz$$

Since $\mu_{2}=1/3$ for the uniform shares and $\mu_{2}=1$ for extreme inequality, define

(A2) $$G_{2}=\frac{3\mu_{2}-1}{2}$$

To derive the share function from the second moment, use

$$z^{2}=\frac{P_{2}(z)}{6\sqrt{5}}+\frac{\sqrt{3}P_{1}(z)}{6}+\frac{P_{0}(z)}{3}$$

(A3) $$\mu_{2}=\int \left[\frac{P_{2}(z)}{6\sqrt{5}}+\frac{\sqrt{3}P_{1}(z)}{6}+\frac{P_{0}(z)}{3}\right]\sum_{n=1}^{N}a_{n}P_{n}(z)dz=\left[\frac{a_{2}}{6\sqrt{5}}+\frac{a_{1}}{6}+\frac{a_{0}}{3}\right]$$

The Lagrangian method subject to the second moment produces

(A4) $$s_{2}(z)=C\exp\left(dz^{2}\right)$$

For the d selected, C is found through normalization. Choose d such that the estimated second moment is the same as the observed second moment.

### Appendix B.2. Derivation of an Inequality Measure and Corresponding Share Function from the Given Third Moment

If only the third moment is utilized,

(A5) $$\mu_{3}=\int z^{3}s(z)\mathrm{dz}\approx \int z^{3}s_{N}(z)\mathrm{dz}=\int z^{3}\sum_{n=1}^{N}a_{n}P_{n}(z)dz$$

Since $\mu_{3}=1/4$ for the uniform shares and $\mu_{3}=1$ for extreme inequality, define

(A6) $$G_{3}=\frac{4\mu_{3}-1}{3}$$

To derive the share function from the third moment, use

$$z^{3}=\frac{P_{3}(z)}{20\sqrt{7}}+\frac{\sqrt{5}P_{2}(z)}{20}+\frac{3\sqrt{3}P_{1}(z)}{20}+\frac{P_{0}(z)}{4}$$

(A7) $$\begin{array}{ll} \mu_{3} & =\int \left[\frac{P_{3}(z)}{20\sqrt{7}}+\frac{\sqrt{5}P_{2}(z)}{20}+\frac{3\sqrt{3}P_{1}(z)}{20}+\frac{P_{0}(z)}{4}\right]\sum_{n=1}^{N}a_{n}P_{n}(z)dz\\ & =\left[\frac{a_{3}}{20\sqrt{7}}+\frac{\sqrt{5}a_{2}}{20}+\frac{3\sqrt{3}a_{1}}{20}+\frac{a_{0}}{4}\right] \end{array}$$

The Lagrangian method subject to the third moment produces

(A8) $$s_{3}(z)=F\exp\left(gz^{3}\right)$$

For the chosen g, F is found with normalization. Choose g such that the estimated third moment is the same as the observed third moment.

### Appendix B.3. Derivation of an Inequality Measure and Corresponding Share Function from the Given Square Root Moment

If only the square root moment is utilized,

(A9) $$\mu_{0.5}=\int \sqrt{z}s(z)\mathrm{dz}\approx \int \sqrt{z}s_{N}(z)\mathrm{dz}=\int \sqrt{z}\sum_{n=1}^{N}a_{n}P_{n}(z)dz$$

Since $\mu_{0.5}=2/3$ for the uniform shares and $\mu_{0.5}=1$ for extreme inequality, define

(A10) $$G_{sqrt}=3\mu_{0.5}-2$$

To derive the share function, use the Lagrangian method subject to the square root moment, which produces

(A11) $$s_{sqrt}(z)=N\exp\left(m\sqrt{z}\right)$$

For the chosen m, N is found with normalization. Choose m such that the estimated square root moment is the same as the observed square root moment.

### Appendix B.4. Derivation of an Inequality Measure and Corresponding share Function from the Given Exponent MOMENT

If only the exponent moment is utilized,

(A12) $$\psi =\int e^{z}s(z)\mathrm{dz}\approx \int e^{z}s_{N}(z)\mathrm{dz}=\int e^{z}\sum_{n=1}^{N}a_{n}P_{n}(z)dz$$

Since ψ=e−1 for the uniform shares and ψ=e for extreme inequality, define

(A13) $$G_{\exp}=\psi +1-e$$

The Lagrangian method subject to the exponent moment produces

(A14) $$s_{\exp}(z)=H\exp\left[\exp\left(kz\right)\right]$$

For the chosen k, H is found with normalization. Choose k such that the estimated exponent moment is the same as the observed exponent moment.

Figure A1 compares estimated shares from various candidates. Only the LBGC measure indicates a good approximation while the other candidate measures seemingly fit less well. Figure A2 shows another comparison. The LBGC distribution approximates the very poor and rich groups well. Figure A3, Figure A4, Figure A5, Figure A6, Figure A7, Figure A8 and Figure A9 compare estimated shares for the bottom 5% share, 20% share (Q1), next 20% share (Q2), next 20% share (Q3), next 20% share (Q4), top 20% share (Q5), and the top 5% group shares. The LBGC method indicates a robust performance for the bottom 5% of income earners, Q1, Q2, and for the top 5% share, but for Q3, Q4, and Q5, the other moment-based measures indicate a better performance. Interestingly, the performance of the actual Gini itself was relatively weak. In Figure A6, the square root model and the actual Gini model did a bit better than the logit model across the third quintile, the Q3 region. The other models such as the exponent model, the second moment model, and the third moment model showed relatively bad performances in this portion of the IDF. In Figure A7, the second moment model seems to be doing well in the fourth quintile, Q4 region. The logit is the next best performer while the square root model and the Gini model indicate relatively poorer performances. In Figure A8, the Gini model, the exponent moment model, and the second moment model performed better than the logit model for the upper portions of the IDF. In Figure A9, the logit model showed a strong performance that was far better than the other Gini variants. In particular, the Gini model and the square root model performed worse than the other models over the fourth quintile. In summary, the logit model demonstrated the best performance for the bottom 5% share of income earners and for the Q1, Q2, and top 5% share income shares. For the other regions of the IDF, the logit model performed as the second or the third highest ranking measure. In other regions, the square root model was strong for Q3, the second moment model was strong for Q4, and the third moment model was the best for Q5.

To summarize the overall fitting performance of these models for the period from 2000 to 2016, we examined how observed income shares have changed from the CPS data over time and compared those observed shares to the estimated shares from our underlying projections. We once again found that the sum of squared residuals produced by various models produced very clear results. For example, the estimated share based on the observed Gini model was presented in Equation (10).

$$RSS_{Gini}(t)={\left[{\mathrm{Observed}\;\mathrm{share}(t)}_{i}-{\mathrm{Gini}\;\mathrm{Estimated}\;\mathrm{share}(t)}_{i}\right]}^{2}$$

Similarly, the estimated shares using the second moment, third moment, square root moment, exponent moment, and logit type moment are presented in (A4), (A8), (A11), (A14) and (17). Year 2013 was somewhat of an anomalous year with more income inequality than prior years or years that followed. The share of income of the richest 1% of earners was 29% in 2013. In comparison, the richest 1% share of 2015 was 23%. Figure A10 shows the logit type moment has an absolute advantage relative to the other moment projections over the entire IDF. By focusing only on results produced by the Gini coefficient, we are throwing out other information contained in the nonlinear part of the underlying IDF, as we utilized only the first moment projection of the share function. In comparison, the logit type projection removed a significant portion of the estimation error.
